# Calypso: a user-friendly web-server for mining and visualizing microbiome–environment interactions

**DOI:** 10.1093/bioinformatics/btw725

**Published:** 2016-12-13

**Authors:** Martha Zakrzewski, Carla Proietti, Jonathan J Ellis, Shihab Hasan, Marie-Jo Brion, Bernard Berger, Lutz Krause

**Affiliations:** 1 QIMR Berghofer Medical Research Institute, Brisbane, QLD, Australia; 2 The University of Queensland Diamantina Institute, Brisbane, QLD, Australia; 3 Nestle Research Center, Vers-chez-les-Blanc, Lausanne, Switzerland

## Abstract

Calypso is an easy-to-use online software suite that allows non-expert users to mine, interpret and compare taxonomic information from metagenomic or 16S rDNA datasets. Calypso has a focus on multivariate statistical approaches that can identify complex environment-microbiome associations. The software enables quantitative visualizations, statistical testing, multivariate analysis, supervised learning, factor analysis, multivariable regression, network analysis and diversity estimates. Comprehensive help pages, tutorials and videos are provided via a wiki page.

**Availability and Implementation:**

The web-interface is accessible via http://cgenome.net/calypso/. The software is programmed in Java, PERL and R and the source code is available from Zenodo (https://zenodo.org/record/50931). The software is freely available for non-commercial users.

**Supplementary information:**

Supplementary data are available at *Bioinformatics* online.

## 1 Introduction

We present the web-application Calypso, a powerful, yet easy-to-use tool for the higher-level analysis of microbial community composition data (e.g. Ainsworth *et al.*, 2015; Cantacessi *et al.*, 2014; Swe *et al.*, 2014). The software has a focus on multivariate methods and allows the analysis of bacterial, archaeal, viral and eukaryotic communities. Several software packages are already available for the analysis and visualization of metagenomic datasets (Arndt *et al.*, 2012; Kristiansson *et al.*, 2009; McMurdie and Holmes, 2013; Parks *et al.*, 2014; Paulson *et al.*, 2013; Sanli *et al.*, 2013). Compared to existing tools, Calypso is unique by providing access to an extensive range of data-mining methods via an easy-to-use web-interface (Tables S1–S3, Fig. S11). The software can easily be explored using a demo project.

## 2 Summary of features

### 2.1 Input files and output formats

As input, Calypso requires a *counts file* providing taxonomic assignments of metagenomic (or 16S rDNA) sequences and a *metadata file* providing meta-information for each sample. An optional matrix of pair-wise community distances can be uploaded, including UniFrac distances. Various file formats are supported, including the common biom-format, which allows direct upload of pre-processed files generated by other analysis pipelines, such as QIIME, mothur, MG-RAST or MetaPhlAn. Uploaded data can be normalized and transformed to account for the generally non-normal distribution of microbial community composition data. Publication-quality images can be generated in either PNG, PDF or SVG format.

### 2.2 Quantitative representations

**Fig. 1:**
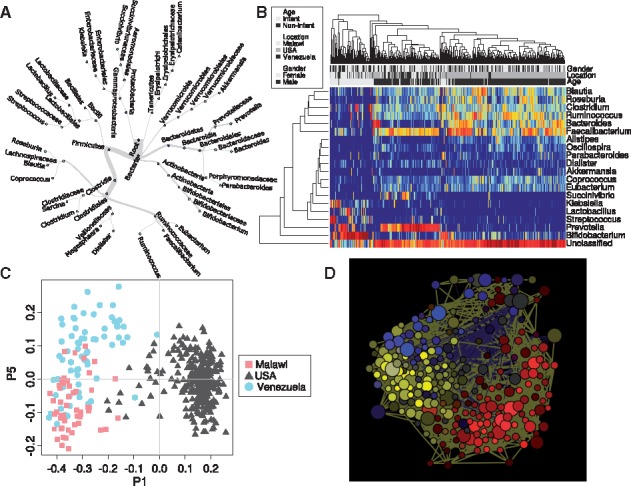
Analysis of intestinal 16S rDNA data in Calypso. (**A**) Interactive trees visualize hierarchical structures in microbial communities. Edges depict the relative abundance of the corresponding taxon. (**B**) Hierarchical clustering of microbial community profiles. (**C**) Principal Coordinates Analysis (PCoA) of intestinal microbiota of subjects from Malawi, USA and Venezuela. (**D**) Network analysis describing positive (yellow edges) and negative associations (blue edges) between bacterial taxa. Nodes are highlighted based on association between OTU abundance and geographic location (red: USA, blue: Venezuela, yellow: Malawi)

Microbial composition data is presented as heatmap, bubble plot, scatter plot, strip chart, bar chart and boxplot. Calypso implements a newly developed module for visualizing hierarchical relationships as interactive dendrograms or interactive radial trees (Fig. 1A, Supplementary Text). Hierarchical relationships can further be explored using Krona charts.

### 2.3 Cluster analysis and sample ordination

Unsupervised clustering of microbial community profiles is achieved by hierarchical clustering (Fig. 1B). Heatmaps can be fine-tuned for components such as the colour palette, trimming of outliers and the centre value of the colour palette. Community composition data is ordinated by principal components analysis, principal coordinates analysis (PCoA) (Fig. 1C), and non-metric multidimensional scaling.

### 2.4 Microbiome–environment associations

Associations between microbial community composition and multiple environmental variables can be identified using a wide range of multivariate methods, including redundancy analysis, canonical correspondence analysis, and permutational MANOVA. Abundance of individual taxa is compared by standard parametric and non-parametric tests and using tests specifically developed for counts data (DESeq2, ANCOM and ALDEx2). Calculated *P*-values are adjusted for multiple testing. Abundance of individual taxa can be associated with multiple biological conditions or confounding factors using multiple linear regression. Mixed effect regression models are used for the analysis of repeated measurements to distinguish between group-specific effects (e.g. case/control) and subject-specific effects. Additionally, feature selection methods facilitate selection of the optimal subset of taxa predictive of an outcome of interest, including step-wise linear regression, LASSO regularized regression and random forest.

### 2.5 Network analysis

A newly developed network module allows the identification of mutual exclusive bacteria and clusters of co-occurring bacteria (Fig. 1D). Taxa are represented as nodes, taxa abundance as node size, and edges depict positive (yellow) and negative (blue) associations. Nodes can be coloured by the phylum or family of the represented bacterial taxon or based on their association to environmental variables. Networks are generated by first computing associations between taxa using Pearson’s correlation. The resulting pairwise correlations are used to ordinate nodes in a two dimensional plot by PCoA. In this way, correlating nodes are placed in close proximity and anti-correlating nodes are placed at distant locations. Nodes of correlating taxa are connected by edges.

### 2.6 Analysis of microbial diversity

Multiple metrics for measuring microbial alpha diversity are provided, including Shannon’s index, evenness, richness, Simpson’s index, Chao 1 and Fisher’s Alpha. Community richness is estimated by rarefaction analysis to account for differences in sample sizes. Complex associations between microbial diversity and multiple explanatory variables are examined by multiple linear regression.

## 3 Conclusions

Calypso provides an easy-to-use statistical and visualization toolbox that allows rapid, robust and thorough analyses of compositional information from microbial datasets. Customized figures of publication-quality can be generated without requiring any programming knowledge.

## Supplementary Material

Supplementary Data
