# Supplementary material for: Calypso: a user-friendly web-server for mining and visualizing microbiome–environment interactions
Source: Bioinformatics. 2016 Dec 13;33(5):782–3. doi: 10.1093/bioinformatics/btw725 (PMC5408814; doi:10.1093/bioinformatics/btw725)
Supplement: Supplementary Data [file btw725_supp.zip › Calypso_Bioinformatics_SupplementaryText.pdf]

Supplementary Material

Calypso: A User-Friendly Web-Server for Mining and Visualizing Microbiome-Environment Interactions.

Martha Zakrzewski[1], Carla Proietti[1], Jonathan J. Ellis[2], Shihab Hasan[1,2], Marie-Jo Brion[2], Bernard Berger[3], Lutz Krause[1,2]

[1]QIMR Berghofer Medical Research Institute, Brisbane, QLD 4006, Australia, [2]The University of Queensland Diamantina Institute, Brisbane, QLD 4102, Australia, [3]Nestle Research Centre, Vers-chez-les-Blanc, Lausanne, Switzerland

The comprehensive and informative analyses of microbiota composition is often hampered by the need for generally high-level computational skills to use the existing (multiple) software tools, by their poor flexibility for data-mining at different taxonomic levels, by their limited selection of statistical methods compared to the range available in ecology (Buttigieg and Ramette, 2014; Dinsdale, et al., 2013; Ramette, 2007), and by the lack of data visualization. These limitations in the existing available software packages are slowing down scientific progress in microbial ecology, despite the explosion of sequencing data.

The web-application Calypso is a powerful, yet easy-to-use tool for the higher-level analysis of microbial community composition data (e.g. (Ainsworth, et al., 2015; Cantacessi, et al., 2014; Dewar, et al., 2014; Dewar, et al., 2014; Giacomin, et al., 2015; Plieskatt, et al., 2013; Reis, et al., 2013; Sarker, et al., 2016; Smith, et al., 2014; Swe, et al., 2014; Umu, et al., 2015; Zhang, et al., 2014).

Calypso input files

As input, Calypso requires a *counts file* providing taxonomic assignments of metagenomic (or 16S rDNA) sequences and a *metadata file* providing meta-information for each sample. Additionally, an optional matrix of pair-wise community distances can be uploaded, which facilitates data analysis using the UniFrac metric. For example, UniFrac distance matrices computed with the popular QIIME pipeline can directly be imported into Calypso.

Metadata file

The metadata file consists of a simple text file in comma- or tab-separated format which provides meta-information for each sample (Figure S1A). Meta-information includes: (i) individual identifiers, which are used for paired analysis (e.g. paired t-

test), if several samples were collected from the same individual (or environment) during a longitudinal study; (ii) sample groups (e.g. case/control or geography); (iii) an *Include* column specifying if samples should be included or excluded from data analysis. This allows the exclusion of outliers or problematic samples without modifying any data files; and iv) multiple optional explanatory (or environmental) variables, which are used in multivariate data analysis. These can represent variables manipulated by the experimenter (e.g. case/control), potential confounding factors (e.g. age, BMI) or other factors that are potentially associated with community composition. Both numeric and categorical variables are supported.

Counts file

The counts file provides the number of 16S or metagenomic sequences assigned to each taxa or operational taxonomic unit (OTU) (Figure S1B). Various file formats are supported, including the common biom-format, which allows direct upload of pre-processed files generated by other analysis pipelines, such as QIIME (Caporaso, et al., 2010), mothur (Schloss, et al., 2009), MG-RAST (Meyer, et al., 2008) or MetaPhlAn (Segata, et al., 2012). Taxonomic assignments obtained for multiple taxonomic ranks (e.g. phylum, family, genus, OTU) can be combined in a single counts file.

Calypso output formats

Results in Calypso can be presented as figures, sortable tables, comma-separated text-files, interactive hierarchical trees, or Krona diagrams. Users can exclude samples, filter bacterial groups, select colors and change figure properties (resolution and dimensions). Publication-quality images can be generated in either PNG, PDF or SVG format. SVG is a XML-based vector graphics format and images in this format can be edited in vector graphics editors, such as Inkscape. This allows post-processing of generated figures to change colors and font sizes, or adding additional labels or features.

Calypso Data-Mining and Statistical Analysis

Data normalization and transformation

Calypso provides various transformation methods to account for the generally non-normal distribution of microbial community composition data. To render the data

suitable for analysis by standard statistical procedures, community profiles can be transformed in Calypso by log, total sum normalization (TSS), asinh, square root, quantile normalization, and variance stabilization and normalization for microarray data (vsn). Sequencing based community profiling yields a special data type called "compositional data", which is characterized by specific intrinsic properties that can deteriorate statistical analysis. In particular, the measured relative abundance of microbial taxa depends on the abundance of all other taxa. To remove the non-independence of relative bacterial abundance, Calypso facilitates data transformation by centered log ratio, which is one of the most widely used transformations for compositional data. The effects of transformation and normalization methods on the data distribution can be visualized in Calypso using boxplots and scatterplots.

Quality control

Calypso enables the implementation of standardized quality control procedures for microbiome data. The distribution of sequences per sample can be plotted as a histogram and potentially problematic samples (outliers) can be detected by hierarchical clustering, principal components analysis (PCA), or principal coordinates analysis (PCoA). The coverage of the underlying microbial communities by metagenomic sequence reads can be estimated by rarefaction analysis. In a rarefaction analysis, microbial sequences are randomly drawn from each sample. For each subsample, the number of observed species is counted and plotted as a function of the number of sampled sequences. The slope of the rarefaction curve indicates if the underlying microbial community is well represented by the sequence data.

Quantitative representation of microbial composition data

Calypso provides a powerful toolbox for presenting microbial composition data quantitatively, including hierarchical trees (Figure S2), heatmaps, bubble plots, scatter plots, stripcharts, bar charts, and boxplots (Figures S3, S4). Hierarchical relationships can be depicted using interactive Krona plots (Ondov, et al., 2011) and taxonomic trees. Krona plots allow assessment of the hierarchical structure of microbial communities using interactive pie charts (Figure S2B). Taxonomic trees visualize hierarchical relationships either as interactive dendrograms (Figure S2A) or interactive radial trees (Figure 2A). Bar charts can be added to depict the abundance of each node (taxa) in each sample (Figure S1A).

Cluster analysis and sample ordination

In Calypso, the unsupervised grouping of samples with similar community composition into clusters is achieved by hierarchical clustering, visualized either as a dendrogram (Figure S5A) or heatmap (Figure S5B). Calypso provides powerful heatmaps for cluster identification, to visualize microbial community composition, and for identifying associations between community composition and the environment. Heatmaps can be fine-tuned according to user preferences, for components such as the color pallet, trimming of outliers, and the center value of the color pallet. Meta information for each sample is displayed as color bars on top of heatmaps.

Calypso provides several ordination methods that allow visualization of community composition data as 2D plots. Samples can be ordinated by the unsupervised methods principal component analysis (PCA), principal coordinates analysis (PCoA), detrended correspondence analysis (DCA), and non-metric multidimensional scaling (NMDS) (Figures S6). All of these pattern-discovery methods are commonly used in microbial ecology for identifying clusters of samples with similar community composition and for identifying potentially problematic samples (outliers).

Identification of microbiome-environment associations

In Calypso, associations between microbial community composition and a single environmental variable can be identified by testing for homogeneity of variances (PERMDISP2), or by comparing intra-group and inter-group community distances using Anosim. Additionally, a wide range of multivariate methods is provided. These are powerful techniques for identifying complex environment-microbiota associations, in which differences in microbial composition can be attributed to multiple variables. Calypso implements the supervised multivariate methods redundancy analysis (RDA), canonical correspondence analysis (CCA), and permutational manova (PERMANOVA/Adonis). These methods test if variance in community composition can be explained by variance in multiple explanatory variables. A separate p-value is provided for each included variable. RDA is widely used in ecology and summarizes linear relationships between components of the community composition matrix and a set of explanatory variables (Buttigieg and Ramette, 2014). CCA, PERMANOVA and Adonis rely on the comparison of community dissimilarities and can be run on UniFrac distances or using dissimilarity indices, such as the Bray-Curtis, Jaccard, Yue & Clayton, or Chao indices.

Network analysis

Calypso enables network analysis for identifying co-occurring bacteria, mutual exclusive bacteria and clusters of co-occurring bacteria (Figure 2D). Taxa and explanatory variables are represented as nodes, taxa abundance as node size, and edges represent positive and negative associations. Nodes can be colored by the phylum or family of the represented bacterial taxon. Alternatively, nodes (taxa) can be colored based on their association with selected environmental variables. Taxa abundances are associated with environmental variables using Pearson's correlation. Nodes are then colored based on the strength of the association with each selected environmental variable. Networks are generated by first computing associations between taxa using the Pearson's correlation index or Spearman's rho. The resulting pairwise correlations are converted into dissimilarities and then used to ordinate nodes in a two dimensional plot by PCoA. In this way, correlating nodes are placed in close proximity and anti-correlating nodes are placed at distant locations in the network. Subsequently, nodes of positively or negatively correlating taxa are connected with yellow/green and blue edges, respectively. Networks are presented as static image or dynamic plots. Dynamic networks have been realized using the Javascript D3.js library. Nodes can be connected with edges based on three different criteria:

(i) Nodes are connected by an edge if their absolute Pearson's correlation or Spearman's rho is above a selected threshold. Alternatively, the significance of Pearson's or Spearman's correlation can be measured and correlations with $p<0.05$ are presented by an edge.

(ii) LASSO regression. Iteratively, the abundance of each taxon is regressed on all remaining taxa using LASSO regression and the most relevant associations are identified. Relevance is assessed by 10-fold cross-validation. Significantly associated taxa are connected be edges. Edge width represent the coefficients learned by the final regression model.

(iii) We have implemented a new ensemble method based on multiple similarity/dissimilarity measures. This method combines Bray-Curtis dissimilarities with Pearson's correlation and Spearman's rho. For each similarity/dissimilarity measure significance of correlation is computed. P-values for Bray-Curtis dissimilarities are computed by 1000-fold permutation. P-values obtained for the multiple similarity/dissimilarity

measures are then combined using the Simes method and corrected for multiple testing by False Discovery Rate (FDR).

(iv) Significant associations (FDR < 0.05) are presented as edge.

Identification of associations between the environment and abundance of individual taxa

Abundance of individual taxa can be compared in Calypso using parametric tests (Anova, nested Anova, t-test, paired t-test, Bayesian t-test) or non-parametric tests (Wilcoxon-rank test and Kruskal-Wallis test). Additionally, differences in taxa abundances can be identified using tests specifically developed for counts data: DESeq2 (Love, et al., 2014), ANCOM (Mandal, et al., 2015), and ALDEx2 (Fernandes, et al., 2014). Calculated p-values are adjusted for multiple testing by Bonferroni correction and False-Discovery-Rate (FDR).

Complex microbiome-environment interactions can also be examined using multiple linear regression (Fig. S7). These are powerful techniques that facilitate identification of associations between individual taxa and multiple explanatory variables. Multivariable paired data can be analyzed by mixed effect regression, which incorporates the paired variable (e.g. subject, animal or cage) as a random effect and other explanatory variables (e.g. case/control or treatment) as fixed effects. These methods can distinguish between group-specific effects (e.g. case/control) and subject or cage-specific effects.

Analysis of microbial diversity

Calypso provides multiple metrics for measuring microbial alpha diversity, including Shannon index, evenness, richness, Simpson index, Chao 1, and Fisher’s Alpha. Community richness is estimated by rarefaction analysis to account for differences in sample sizes. Complex associations between microbial diversity and multiple explanatory variables are identified by multiple linear regression (Fig. S7). Calypso further supports diversity analysis using mcpHill (Pallmann, et al., 2012), which simultaneously investigates several diversity measures by unifying them in one of the same mathematical family of indices.

Biomarker discovery

Calypso features a module for biomarker discovery, which identifies bacterial taxa predictive of an outcome of interest (e.g. responders/non-responders, disease/healthy, high risk/low risk). The discriminatory power of microbial community profiles to distinguish between two sample groups is characterized by a Support Vector Machine evaluated by leave-one-out cross-validation. The classification performance is described by overall accuracy, sensitivity and specificity. The discriminatory power of individual taxa can be assessed by area under the ROC curve (AUC), odds ratio, delta (difference in means in units of standard deviation), and fold change (Table S4).

Advanced feature selection methods in Calypso facilitate selection of the optimal subset of taxa predictive of an outcome of interest. These methods further allow the identification of relevant taxa associated with an explanatory variable. In Calypso, feature selection methods are based on the premise that many taxa are either redundant (highly correlated) or irrelevant, and can thus be removed without much loss of information. Calypso implements the widely used feature selection methods step-wise linear regression, LASSO regularized regression, random forest and LDA Effect Size (LEfSe). LASSO performs both feature selection and regularization to prevent overfitting. Taxa selected by stepwise regression or LASSO regularized regression are presented in Calypso as bar charts, where bars depict the importance of each taxa (Akaike information criterion [AIC] of the model if the taxa was dropped from the model or absolute of the t-statistic, respectively). Random forest identifies the subset of most relevant features by constructing a collection of decision trees. Variance is controlled by constructing trees incorporating only a random subset of the features, which in turn avoids overfitting. In Calypso, the results of the random forest analysis are presented as bar chart, where bars represent the relative importance of taxa, as estimated by random permutation. LEfSe determines the taxa most likely to explain differences between classes by coupling standard tests for statistical significance with additional tests encoding biological consistency and effect relevance.

Visualization of hierarchical relationships

Calypso features a module for the interactive visualization of taxonomic information in a hierarchical structure. Hierarchical data can be visualized using Krona charts or

using newly developed dynamic dendrograms and radial trees. Dendrograms and radial trees have been implemented using the tree layout provided in the Javascript D3.js library and interactivity has been realized using Javascript. Users can customize the hierarchical trees by displaying only selected nodes, adding barcharts representing taxa abundances and by changing the text size.

Calypso application example

To demonstrate Calypso's abilities, we re-analyzed a fecal 16S rDNA dataset from 531 individuals recently published by Yatsunenko (Yatsunenko, et al., 2012). The individuals were in age range of 3 months to 83 years and from metropolitan regions in the United States, rural communities in Malawi and Amerindian villages in Venezuela. PCoA separated subjects by age and geography (Figures 1C and S6A). These findings were supported by supervised multivariate analysis by RDA and CCA, which found significant associations between the composition of the intestinal microbiota and age and geographic location, and additionally with kinship and gender ($p<0.05$, Table S5). This multivariate analysis of community profiles included age, geographic location, kinship and gender as explanatory variables. Inline with these results, a network analysis found two clearly distinct microbial clusters associated with age and location (Figures 2D). Also microbial diversity (Shannon index and richness) was influenced by age and location, as revealed by multivariable regression (Figures S7A-B). In this analysis, microbial diversity (Shannon index or richness) was regressed on age, location, gender and kinship. Adults from the US showed a lower bacterial richness when compared to adults from rural communities (Figure S9B).

PCoA, NMDS, RDA and hierarchical clustering showed that individuals of rural countries (Malawi and Venezuela) had a similar intestinal microbiota. Contrastingly, the intestinal microbiota was considerably different between populations from rural countries and the USA (Figures S6A-C, S8A-B). Fecal microbial profiles were predictive of geographic location and a Support Vector Machine evaluated by leave-one-out cross validation was able to predict the environment (metropolitan vs. rural) with 95% accuracy. This analysis supports our findings of an environment specific microbiome. Fifteen genera showed differential abundance between rural and metropolitan populations (Bonferroni corrected p-values in range 10^{-57}-0.00011) (Table S4). Of the dominant genera, prevalence of *Prevotella*, *Ruminococcus* and

Bacteroides were associated with geographic location (Figures S4). Seven genera were able to discriminate between rural and metropolitan populations with high accuracy with an AUC in range 0.8-1.0. The most discriminative genera was *Succinivibrio* (AUC=1), which showed an average relative abundance of 3.45% in rural populations (>3 years), but which was not detected in the metropolitan group (Table S4).

The adult microbiota was more stable and similar than the infant microbiota. In a PCoA, adult microbial samples from the same geographic location clustered tightly together, whereas the infant microbiota showed high inter-individual variability (Figure S6A). Accordingly, microbial profiles of infants showed a significantly higher pair-wise distance than the microbiota of adults (Figure S8C). A number of genera were significantly differentially abundant between age ranges (Figure 8D). Multivariable regression adjusted for location and gender identified a significant decrease in *Bifidobacteria* (FDR<10^{-30}) and *Lactobacillus* (FDR=0.003) and an increase in *Faecalibacterium* (FDR=0.009) with increasing age (Figure S7C-E). In this analysis, relative bacterial abundance was regressed on age, location and gender. During the first three years of life, the intestinal microbiota evolves towards an adult-like state (Table S6 and S7). We observed a significant increase of microbial diversity (Shannon index), richness and evenness with age by univariate statistics (Figure S9), which was still significant when correcting for gender and location by multivariable regression (p<0.0001) (Figure S7A-B).

Gut microbial profiles of USA infants further clustered by diet (breast-fed vs. formula-fed) (Figure S6D) (p=0.014, Anosim). Breast-fed infants had a significantly higher relative abundance of *Bifidobacterium*, *Clostridium*, *Lactobacillus* and *Bacillales* species and lower relative abundance of *Ruminococcus* and other *Clostridium* species (p<0.05, Anova) (Figure S10A). Breast feeding was further associated with lower microbial diversity (Shannon index) and lower microbial richness (Figure S10B-C).
These results are consistent with the main findings presented by Yatsunenko (Yatsunenko, et al., 2012), and demonstrate that Calypso enables accurate and rapid in-depth analysis of complex 16S rDNA datasets. Notably, with Calypso the entire dataset could be comprehensively analyzed in a few hours. Generated high-quality figures can be directly used for publication (e.g. Ainsworth, et al., 2015; Cantacessi,

et al., 2014; Dewar, et al., 2014; Dewar, et al., 2014; Giacomin, et al., 2015; Plieskatt, et al., 2013; Reis, et al., 2013; Sarker, et al., 2016; Smith, et al., 2014; Swe, et al., 2014; Umu, et al., 2015; Zhang, et al., 2014).

Data and Methods

Calypso implementation

Calypso is programmed in Java, PERL and R and is based on the GMine framework (Proietti, et al., 2016). The Calypso web-frontend is implemented in Java using the JavaServer Faces architecture and the backend is implemented in Perl and the R statistical programming environment using existing packages and newly developed modules. The software is running on a virtual server of the National eResearch Collaboration Tools and Resources (Nectar) cloud. A wiki page providing extensive help and a tutorial, has been created using the MediaWiki package.

Example dataset

To demonstrate the utility of our software, we used Calypso to re-analyze a fecal 16S rDNA dataset previously published by Yatsunenko (Yatsunenko, et al., 2012). The number of 16S sequences assigned to each bacterial taxa and metadata for each sample of the Yatsunenko et al. dataset were downloaded from the Gordon laboratory web-page at Washington University. Metadata included age, geographic location, gender, family id and diet during infancy. All analysis in Calypso were based on total-sum normalized (TSS) 16S data. All analyses were carried out on genus level. Microbial community composition was associated with multiple environmental variables by the multivariate statistical methods redundancy analysis (RDA), canonical correspondence analysis (CCA) and Adonis. In this analysis, age, geographic location, kinship and gender were included as explanatory variables. Additionally, microbial community composition was associated with environmental variables using the univariate method Anosim. In this analysis, Anosim was run separately for each environmental variable (age, location, kinship and gender). Microbial diversity (Shannon index and richness) was associated with environmental variables by multivariable regression of the form: diversity = age + location + gender + kinship. A Support Vector Machine (SVM) was trained on the microbial community profiles to predict metropolitan vs. rural environments and evaluated by leave-one-out cross-validation. Microbial genera were associated with age by multiple linear regression,

including geographic location and gender as possible confounding factors. The regression models had the form: genus abundance = age + geographic location + gender. Microbial diversity (Shannon index) was associated with age by multivariable regression, including geographic location and gender as possible confounding factors: Shannon index = age + gender + location.

References

Ainsworth, T.D., et al. (2015) The coral core microbiome identifies rare bacterial taxa as ubiquitous endosymbionts, The ISME journal, 9, 2261-2274.

Buttigieg, P.L. and Ramette, A. (2014) A guide to statistical analysis in microbial ecology: a community-focused, living review of multivariate data analyses, FEMS microbiology ecology, 90, 543-550.

Cantacessi, C., et al. (2014) Impact of experimental hookworm infection on the human gut microbiota, The Journal of infectious diseases, 210, 1431-1434.

Caporaso, J.G., et al. (2010) QIIME allows analysis of high-throughput community sequencing data, Nature methods, 7, 335-336.

Dewar, M.L., et al. (2014) Interspecific variations in the faecal microbiota of Procellariiform seabirds, FEMS microbiology ecology, 89, 47-55.

Dewar, M.L., et al. (2014) Influence of fasting during moult on the faecal microbiota of penguins, PloS one, 9, e99996.

Dinsdale, E.A., et al. (2013) Multivariate analysis of functional metagenomes, Frontiers in genetics, 4, 41.

Fernandes, A.D., et al. (2014) Unifying the analysis of high-throughput sequencing datasets: characterizing RNA-seq, 16S rRNA gene sequencing and selective growth experiments by compositional data analysis, Microbiome, 2, 15.

Giacomin, P., et al. (2015) Experimental hookworm infection and escalating gluten challenges are associated with increased microbial richness in celiac subjects, Scientific reports, 5, 13797.

Love, M.I., Huber, W. and Anders, S. (2014) Moderated estimation of fold change and dispersion for RNA-seq data with DESeq2, Genome biology, 15, 550.

Mandal, S., et al. (2015) Analysis of composition of microbiomes: a novel method for studying microbial composition, Microbial ecology in health and disease, 26, 27663.

Meyer, F., et al. (2008) The metagenomics RAST server - a public resource for the automatic phylogenetic and functional analysis of metagenomes, BMC bioinformatics, 9, 386.

Ondov, B.D., Bergman, N.H. and Phillippy, A.M. (2011) Interactive metagenomic visualization in a Web browser, BMC bioinformatics, 12, 385.

Pallmann, P., et al. (2012) Assessing group differences in biodiversity by simultaneously testing a user-defined selection of diversity indices, Molecular ecology resources, 12, 1068-1078.

Plieskatt, J.L., et al. (2013) Infection with the carcinogenic liver fluke Opisthorchis viverrini modifies intestinal and biliary microbiome, FASEB journal : official publication of the Federation of American Societies for Experimental Biology, 27, 4572-4584.

Proietti, C., et al. (2016) Mining, visualizing and comparing multidimensional biomolecular data using the Genomics Data Miner (GMine) Web-Server, Scientific Reports.

Ramette, A. (2007) Multivariate analyses in microbial ecology, FEMS microbiology ecology, 62, 142-160.

Reis, M.G., et al. (2013) Impact of dietary dairy polar lipids on lipid metabolism of mice fed a high-fat diet, Journal of agricultural and food chemistry, 61, 2729-2738.

Sarker, S.A., et al. (2016) Oral Phage Therapy of Acute Bacterial Diarrhea With Two Coliphage Preparations: A Randomized Trial in Children From Bangladesh, EBioMedicine, 4, 124-137.

Schloss, P.D., et al. (2009) Introducing mothur: open-source, platform-independent, community-supported software for describing and comparing microbial communities, Applied and environmental microbiology, 75, 7537-7541.

Segata, N., et al. (2012) Metagenomic microbial community profiling using unique clade-specific marker genes, Nature methods, 9, 811-814.

Smith, D.J., et al. (2014) Pyrosequencing reveals transient cystic fibrosis lung microbiome changes with intravenous antibiotics, The European respiratory journal, 44, 922-930.

Swe, P.M., et al. (2014) Scabies mites alter the skin microbiome and promote growth of opportunistic pathogens in a porcine model, PLoS neglected tropical diseases, 8, e2897.

Umu, O.C., et al. (2015) Resistant starch diet induces change in the swine microbiome and a predominance of beneficial bacterial populations, Microbiome, 3, 16.

Yatsunenko, T., et al. (2012) Human gut microbiome viewed across age and geography, Nature, 486, 222-227.

Zhang, L., et al. (2014) Molecular investigation of bacterial communities on intravascular catheters: no longer just Staphylococcus, European journal of clinical microbiology & infectious diseases: official publication of the European Society of Clinical Microbiology, 33, 1189-1198.
