# Supplementary material for: Calypso: a user-friendly web-server for mining and visualizing microbiome–environment interactions
Source: Bioinformatics. 2016 Dec 13;33(5):782–3. doi: 10.1093/bioinformatics/btw725 (PMC5408814; doi:10.1093/bioinformatics/btw725)
Supplement: Supplementary Data [file btw725_supp.zip › Calypso_Bioinformatics_SupplementaryFigures_Tables_REV2.pdf]

Supplementary Table S1: Overview of graphical and statistical methods provided by Calypso.

Graphical

Method	Description
Heat map	Visualize microbial composition, identify sample clusters and explore microbiome-environment associations
Bubble plot	Visualize microbial community composition
Krona plot, hierarchical tree	Explore hierarchical structure of microbial communities
Bar charts, box plots and strip charts	Illustrate taxa abundance and microbial diversity

Statistical

Method	Description
Network analysis	Correlation network showing co-occurring and mutual exclusive taxa
PCoA, PCA, DCA, NMDS	Unsupervised ordination methods used for data clustering and the identification of outliers
Anosim, PERMDISP2	Supervised univariate methods for identifying significant associations between community composition and a single explanatory variable
RDA, Adonis, CCA	Supervised multivariate method for identifying significant associations between microbial community composition and multiple explanatory variables
Partial least squares regression (PLS)	Multivariate method used to identify taxa associated with multiple explanatory variables
(Paired) T-test, (nested) Anova, logistic regression, (paired) Wilcoxon rank test, Kruskal-Wallis test	Identify taxa significantly differentially abundant between sample groups
DESeq2, ANCOM, ALDEx2	Methods specifically developed for counts data. Used for identifying taxa significantly differentially abundant between sample groups.
Multiple linear regression	Identify significant associations between individual taxa and multiple explanatory variables
Support Vector Machine (SVM)	Examine if microbial community composition is predictive of an outcome of interest
Step-wise regression, LASSO regularized regression, random forest	Feature selection methods used for identifying a subset of relevant taxa predictive of an outcome of interest
Shannon index, richness, evenness, Chao 1, ACE, Fisher's Alpha, Simpson index	Quantify microbial alpha diversity
mcpHill	Assess microbiome diversity on multiple indices simultaneously
Jaccard, Bray-Curtis, Yue & Clayton, Chao	Calculate pairwise distances of microbial community profiles
Rarefaction analysis	Estimate coverage of microbial diversity by sequence data

Table S2. Overview of basic features of Calypso and several existing software tools for the analysis of microbial community composition data.

	**Calypso**	**phyloseq**	**ShotgunFunctionalizeR**	**MetagenomeSeq**	**STAMP**	**FANTOM**	**METAGENassist**
**Implementation**	web-based	R package	R package	R package	standalone software	standalone software	web-based
**File format**	Biom, QIIME format tab-separated files, csv	biom, mothur, tab-separated files	tab-separated files	biom format, tab-separated files, QIIME format	biom, MG-RAST, IMG/M, tab-separated files	tab-separated files	csv, common formats (e.g. biom, STAMP, mothur)
**Data normalization**	Yes	Yes	No	Yes	No	No	Yes
**Rarefaction analysis**	Yes	Yes	Yes	Yes	No	No	No
**Diversity analysis**	Yes	Yes	Yes	No	No	No	No
**Quantitative visualizations**	barcharts, bubble plots, heatmap, scatterplot, stripchart	heatmap, barcharts	barcharts	heatmap, abundance plot, bubble plot	barcharts, heatmap	bar graphics, box plot, pie chart, area plot	bar charts, pie charts
**Univariate statistical testing**	(Paired) T-test, ANOVA, nested ANOVA, Bayes ANOVA, (paired) Wilcoxon rank test, Kruskal-Wallis test, DESeq2, ANCOM, ALDEx2, ANOSIM	t-test, paired permutation t-test, ANOVA; hypergeometric test	binomial, hypergeometric and the Gaussian test, poisson test	zero-inflated log-normal mixutre model, zero-inflated gaussian mixture model	Welch's t-test, White's nonparametic t-test, ANOVA, Kruskal-Wallis H-test, Fisher's exact test	Levene's, Bartlet's test, Student's t-test, Welch's t-test, Mann-Whitney U Test	t-test, ANOVA, Mann-Whitney test, Kruskal-Wallis test
**Correction for multiple testing**	Yes	Yes	Yes	Yes	Yes	Yes	No
**Distance-based methods**	Yes	Yes	No	Yes	No	No	No

Table S3. Overview of advanced features of Calypso and several existing software tools for the analysis of microbial community composition data.

	**Calypso**	**phyloseq**	**ShotgunFunctionalizeR**	**MetagenomeSeq**	**STAMP**	**FANTOM**	**METAGENassist**
**Multivariate data analysis**	PCA, PCoA, PLS, RDA, CCA, DCA, Adonis, NMDS, permutational MANOVA and others	DCA, CCA, RDA, DPCoA, NMDSMDS, PCoA and others	No	PCA, MDS	PCA	PCA	PCA, PLS-DA
**Clustering**	Dendrogram, heatmap, SOM	Dendrogram	Dendrogram	Heatmap	Heatmap	Heatmap	Dendrogram, SOM, heatmap
**Regression**	Yes	No	Yes	Yes	No	No	No
**Correlation analysis**	Pearson, Spearman, Kendall correlation; Network analysis, WGCNA, factor analysis, heatmap	Network analysis, scatter plots	No	Correlation heatmap	No	Pearson, Spearman's rank correlation, scatter plots	Correlation heatmap (Pearson, Spearman, Kendall)
**Hierarchical trees**	Yes	Yes	No	No	No	No	No
**Krona plots**	Yes	No	No	No	No	No	No
**Feature selection**	Random forest, LASSO regression, step-wise regression, LEfSe	No	No	No	No	No	Random forest
**Classification**	SVM evaluated by LOOCV	No	No	No	No	No	SVM evaluated by LOOCV

Supplementary Table S4: Biomarker discovery in Calypso to identify genera predictive of geography (rural versus metropolitan populations >3 yr). Abundance of genera were compared by Wilcoxon rank test and adjusted for multiple testing by false discovery rate (FDR) and Bonferroni correction. The area under the curve (AUC) describes the discriminatory power of each genus to distinguish rural from metropolitan populations. Also the odds ratio, the upper and lower confidence intervals of the odds ratio, delta (ratio of difference in means in units of the standard deviation), fold change (ratio of means) and mean relative abundance are given. Only genera with a mean abundance of at least 1% in the rural or USA cohort are shown.

Taxa	P	FDR	Bonferroni	AUC	AUC.UpperCI	AUC.LowerCI	OddsRatio Rural/USA	UpperCI	LowerCI	Delta	FoldChange	Mean Rural	Mean USA
**Succinivibrio**	2.3E-59	5.3E-58	1.5E-57	1	1	1	171529878.3	2.09E+96	0	1.26	5819.78	3.45	0
**Alistipes**	1.8E-39	2.1E-38	1.2E-37	0.95	0.98	0.92	0.03	0.06	0.01	1.66	0.08	0.22	2.84
**Bacteroides**	2.5E-36	1.4E-35	1.5E-34	0.93	0.97	0.89	0.04	0.08	0.01	1.24	0.17	1.43	8.25
**Prevotella**	6.1E-36	3E-35	3.5E-34	0.93	0.95	0.9	30.28	58.66	16.36	1.74	8.67	17.52	2.02
**Lactobacillus**	1.6E-28	4.6E-28	7.5E-27	0.88	0.91	0.84	15.94	69.73	5.15	0.48	38.17	1.79	0.05
**Klebsiella**	2.2E-28	6E-28	1E-26	0.88	0.91	0.84	32.2	110.72	12.3	0.83	22.23	3.2	0.14
**Parabacteroides**	4.8E-24	1.2E-23	2.1E-22	0.85	0.89	0.8	0.04	0.09	0.01	0.92	0.17	0.27	1.64
**Akkermansia**	4.2E-17	8.3E-17	1.5E-15	0.79	0.84	0.74	0.16	0.33	0.07	0.81	0.1	0.12	1.14
**Clostridium**	2.7E-16	5.2E-16	9.3E-15	0.78	0.84	0.72	0.14	0.24	0.08	0.96	0.57	3.42	5.98
**Ruminococcus**	5.8E-15	1.1E-14	1.9E-13	0.77	0.82	0.72	0.15	0.26	0.08	1.06	0.48	3.89	8.07
**Coprococcus**	7.3E-13	1.2E-12	2.2E-11	0.74	0.8	0.69	0.21	0.35	0.12	0.84	0.55	2.04	3.68
**Roseburia**	1.9E-10	3.1E-10	5.4E-09	0.72	0.78	0.66	0.29	0.47	0.17	0.76	0.49	2.75	5.61
**Unclassified**	8.1E-10	1.2E-09	0.00000002	0.71	0.77	0.65	0.31	0.5	0.19	0.79	0.74	21.9	29.59
**Blautia**	1.7E-08	2.5E-08	0.00000038	0.69	0.75	0.63	0.37	0.62	0.22	0.51	0.64	4.32	6.71
**Dialister**	6.3E-06	8.3E-06	0.00011	0.65	0.71	0.6	1.48	2.38	0.91	0.36	1.65	1.73	1.05
**Faecalibacterium**	0.0053	0.0065	0.074	0.6	0.66	0.53	0.55	0.88	0.34	0.38	0.79	8.71	11.01
**Streptococcus**	0.042	0.05	0.47	0.57	0.64	0.5	4.43	9.81	2.07	0.44	5.79	2.42	0.42
**Bifidobacterium**	0.097	0.11	0.77	0.56	0.63	0.48	1.3	2.33	0.71	0.49	2.89	7.57	2.62
**Eubacterium**	0.28	0.3	1	0.46	0.53	0.39	1.04	1.65	0.65	0.11	0.92	3.12	3.38
**Oscillospira**	0.95	0.95	1	0.5	0.57	0.43	1.34	2.15	0.83	0.1	1.1	2.18	1.97

Supplementary Table S5: Association of gut microbiota composition with age, geographic location, kinship and gender using various multivariate and univariate statistical methods (OTU-based). Significant associations are highlighted. Age, geographic location, family were significantly associated with gut microbiota composition. RDA, CCA and Adonis were run in a multivariate manner, including age, geographic location, family id and gender as explanatory variables. Anosim was run separately for age, geographic location, family and gender. RDA: Redundancy Analysis; CCA: Canonical Correspondence Analysis.

	Age	Geographic location	Family	Gender
RDA	**0.001**	**0.001**	**0.001**	**0.006**
CCA	**0.001**	**0.001**	**0.001**	0.132
Adonis	**0.0007**	**0.0007**	**0.0007**	**0.019**
Anosim	**0.001**	**0.001**	**0.001**	0.119

Table S6: Median pairwise distances (Jaccard) of fecal microbial profiles for different age ranges in rural communities. The infant microbiota (<1 and 1-2 years) shows a high variability, reflected by a high median pairwise distance (highlighted in blue). An adult-like fecal microbial composition is reached at the age of 2-4 (highlighted in brown).

	**<1**	**1-2**	**2-4**	**4-18**	**≥18**
**<1**	0.75	0.85	0.91	0.94	0.94
**1-2**	0.82	0.8	0.83	0.84	0.86
**2-4**	0.91	0.83	0.75	0.74	0.76
**4-18**	0.94	0.84	0.74	0.67	0.71
**≥18**	0.94	0.86	0.76	0.71	0.72

.

Table S7: Median pairwise distances (Jaccard) of fecal microbial profiles for different age ranges in subjects from the USA. The infant microbiota (<1 and 1-2 years) shows a high variability, reflected by a high median pairwise distance (highlighted in blue). An adult-like fecal microbial composition is reached at the age of 2-4 (highlighted in brown).

	**<1**	**1-2**	**2-4**	**4-18**	**≥18**
**<1**	0.85	0.92	0.93	0.96	0.96
**1-2**	0.92	0.84	0.79	0.84	0.85
**2-4**	0.93	0.79	0.77	0.8	0.81
**4-18**	0.96	0.84	0.80	0.77	0.77
**≥18**	0.96	0.85	0.81	0.77	0.77

A

Sample ID	Label	Family	Location (Primary Group)	Age group (Secondary group)	Include	Gender	BMI	Age	Location
h208A.1	h208A.1_Malawi_0.032854209	h208	Rural	group1:0	1	Female	11	0.03	Malawi
USinfTw2.1	USinfTw2.1_USA_0.083333333	USinfTw2	Metropolitan	group1:0	1	Male	NA	0.08	USA
USinfTw3.1	USinfTw3.1_USA_0.166666667	USinfTw3	Metropolitan	group1:0	1	Female	NA	0.17	USA
h257B.1	h257B.1_Malawi_0.19	h257	Rural	group1:0	1	Male	16.6	0.19	Malawi
h181A.1	h181A.1_Malawi_0.213552361	h181	Rural	group1:0	1	Male	15.5	0.21	Malawi
USinfTw21.1	USinfTw21.1_USA_0.25	USinfTw21	Metropolitan	group1:0	1	Female	NA	0.25	USA
USinfTw5.1	USinfTw5.1_USA_0.25	USinfTw5	Metropolitan	group1:0	1	Male	NA	0.25	USA
Amz1baby	Amz1baby_Venezuela_0.25	AmzP1	Rural	group1:0	1	Male	NA	0.25	Venezuela
USinfTw7.1	USinfTw7.1_USA_0.333333333	USinfTw7	Metropolitan	group1:0	1	Male	NA	0.33	USA
USinfTw10.2	USinfTw10.2_USA_0.416666667	USinfTw10	Metropolitan	group1:0	1	Male	NA	0.42	USA
USinfTw19.1	USinfTw19.1_USA_0.416666667	USinfTw19	Metropolitan	group1:0	1	Female	NA	0.42	USA
USinfTw9.1	USinfTw9.1_USA_0.416666667	USinfTw9	Metropolitan	group1:0	1	Female	NA	0.42	USA
AmzC1babyF2	AmzC1babyF2_Venezuela_0.417	AmzC1	Rural	group1:0	1	Female	18.2	0.42	Venezuela
h165A.1	h165A.1_Malawi_0.443531828	h165	Rural	group1:0	1	Male	16	0.44	Malawi
USinfTw11.2	USinfTw11.2_USA_0.5	USinfTw11	Metropolitan	group1:0	1	Female	NA	0.5	USA
h209A.2	h209A.2_Malawi_0.563997262	h209	Rural	group1:0	1	Female	16.2	0.56	Malawi
h235B.1	h235B.1_Malawi_0.665297741	h235	Rural	group1:0	1	Male	16.8	0.67	Malawi
USinfTw15.1	USinfTw15.1_USA_0.666666667	USinfTw15	Metropolitan	group1:0	1	Female	NA	0.67	USA
USinfTw16.2	USinfTw16.2_USA_0.75	USinfTw16	Metropolitan	group1:0	1	Male	NA	0.75	USA
h264B.2	h264B.2_Malawi_0.79945243	h264	Rural	group1:0	1	Female	16.2	0.8	Malawi
USinfTw18.1	USinfTw18.1_USA_0.916666667	USinfTw18	Metropolitan	group1:0	1	Male	NA	0.92	USA
h273A.2	h273A.2_Malawi_0.928131417	h273	Rural	group1:0	1	Female	17.5	0.93	Malawi
USchp41Infant	USchp41Infant_USA_1	USchp41	Metropolitan	group2:1	1	Male	19	1	USA
Amz11inf	Amz11inf_Venezuela_1	AmzP_NA	Rural	group2:1	1	Male	15.4	1	Venezuela
AmzC4chldF	AmzC4chldF_Venezuela_1	AmzC4	Rural	group2:1	1	Female	19.3	1	Venezuela
Amz13chld	Amz13chld_Venezuela_1.166666667	AmzP_NA	Rural	group2:1	1	Female	15.8	1.2	Venezuela
Amz14chld	Amz14chld_Venezuela_1.166666667	AmzP_NA	Rural	group2:1	1	Female	15.2	1.2	Venezuela
h144B.1	h144B.1_Malawi_1.2183436	h144	Rural	group2:1	1	Male	14.6	1.22	Malawi
USBld1	USBld1_USA_1.25	USAbld_NA	Metropolitan	group2:1	1	Female	NA	1.25	USA

B

OTU	Header	h278B.2	USygt45.T2	USygt45.T1	Amz7adltF	AmzC13baby	Amz21chld	USygt36.T1	USygt36.T2	h165S	h47M	USygt5.T1
OTU	p__Actinobacteria; g__Bifidobacterium 338437	520804	20584	114912	612	163	1588	34316	96692	254	347	65151
OTU	p__Firmicutes; g__Faecalibacterium 165924	61841	134015	4666	8566	649	22989	118644	22878	52945	19994	202216
OTU	p__Bacteroidetes; o__Bacteroidales 183618	68	121828	23050	129	91	9	281687	184293	1179	2394	76919
OTU	p__Firmicutes; g__Faecalibacterium 174902	5079	45258	496	26161	4430	58961	16404	98261	120895	114176	88816
OTU	p__Firmicutes; g__Roseburia 192252	100774	91010	42211	2532	124	17693	45053	93943	57494	73811	99041

⋮

phylum	Header	h278B.2	USygt45.T2	USygt45.T1	Amz7adltF	AmzC13baby	Amz21chld	USygt36.T1	USygt36.T2	h165S	h47M	USygt5.T1
phylum	Armatimonadetes	0	0	0	0	0	0	0	0	0	0	0
phylum	SC4	0	0	0	0	0	0	0	0	0	0	0
phylum	Cyanobacteria	2756	9	10	2904	131	5022	2	0	33211	13167	0
phylum	TG3	0	0	0	0	0	0	0	0	0	0	0
phylum	Fusobacteria	6	14	0	8065	5	845	1152	11946	104	77	0
phylum	Crenarchaeota	0	0	0	0	0	0	0	0	0	0	0

⋮

genus	Header	h278B.2	USygt45.T2	USygt45.T1	Amz7adltF	AmzC13baby	Amz21chld	USygt36.T1	USygt36.T2	h165S	h47M	USygt5.T1
genus	Unclassified	101804	434468	412427	668830	336304	367145	679799	611360	715365	443062	391083
genus	Faecalibacterium	83725	239719	7604	43788	198681	100211	147731	173942	351686	180946	421967
genus	Prevotella	545430	181373	39	35372	927	268369	1206	105	351688	422518	151450

⋮

family	Header	h278B.2	USygt45.T2	USygt45.T1	Amz7adltF	AmzC13baby	Amz21chld	USygt36.T1	USygt36.T2	h165S	h47M	USygt5.T1
family	Ruminococcaceae	98667	364520	353340	518325	201677	320642	473130	508511	736578	345439	686679
family	Lachnospiraceae	201925	788674	868552	160231	263239	210477	360463	463498	501171	493250	466950
family	Unclassified	49442	174984	98077	201130	115915	135634	438479	263839	156059	129087	149618
family	Prevotellaceae	545430	181380	40	39582	955	268371	1207	105	351691	422518	151450

Figure S1: (A) The meta-information file providing the sample identifier, sample label (shown in generated figures instead of sample identifier), identifiers of individuals, primary and secondary sample groups and a column defining which samples to include in the analysis. Additional, multiple optional explanatory variables can be provided, which are used in multivariate analysis to identify complex microbiome-environment associations. (B) The counts file provides the number of 16S or metagenomic sequences assigned to each taxa or OTU. Counts data for multiple taxonomic ranks can be combined in a single file. The taxonomic rank is provided in the first column, followed by the taxa names and the number os sequences assigned to each sample. Each taxonomic rank has a separate header line.

A

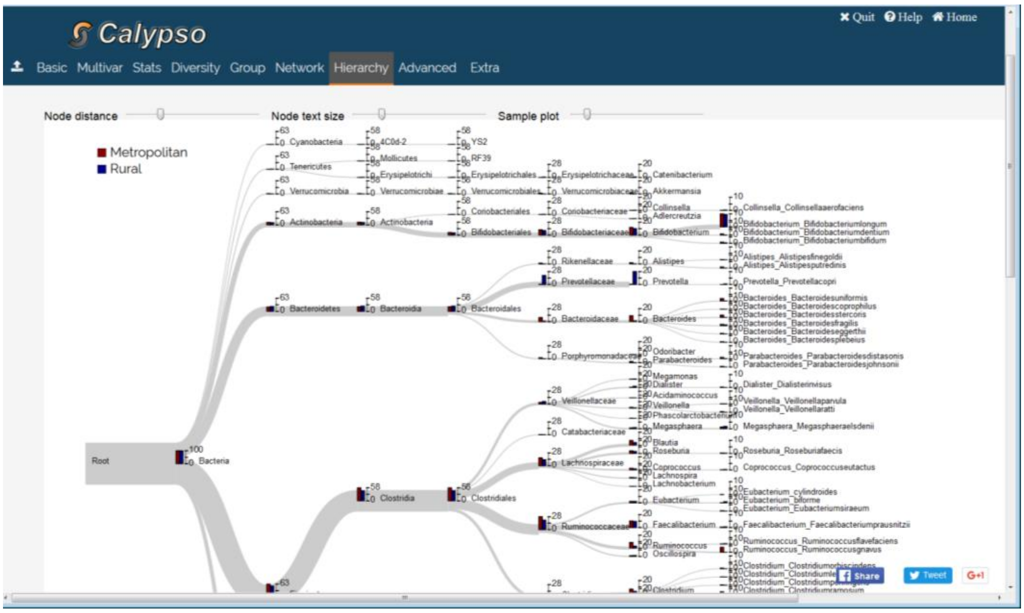

B

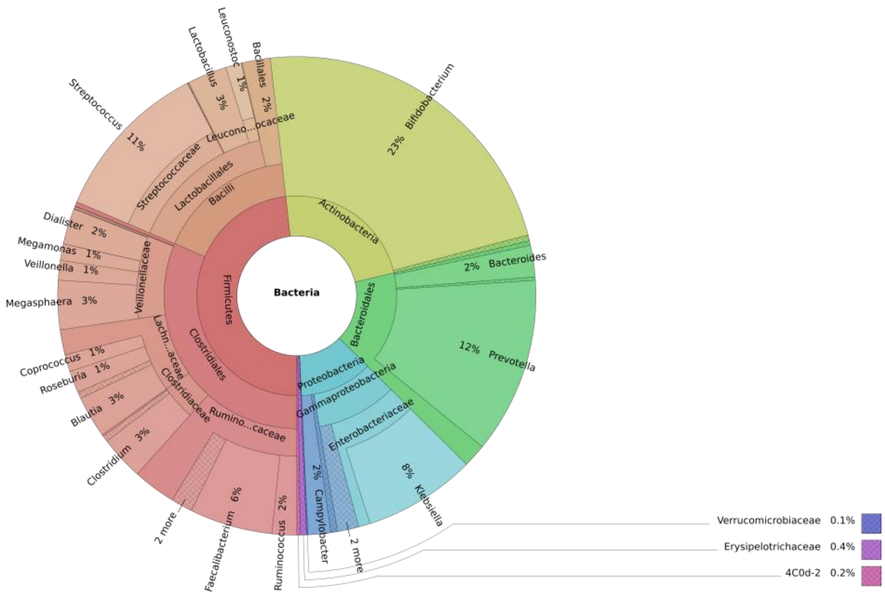

Figure S2: (A) Web-frontend of Calypso. The help link on the top right provides comprehensive help, detailed description and work flows. Shown is an interactive taxonomic tree visualizing the abundance of detected taxa from superkingdom to family rank. Bar charts illustrate the mean relative abundance of taxa in infants and non-infants. Edges widths reflect the relative abundance of each taxon. Only the 14 most abundant families are shown. (B) Krona visualization of the gut microbiota collected from breast-fed. The chart was generated in Calypso using the tool Krona. The genus *Bifidobacteriums* contributes about 25% of the total microbial community.

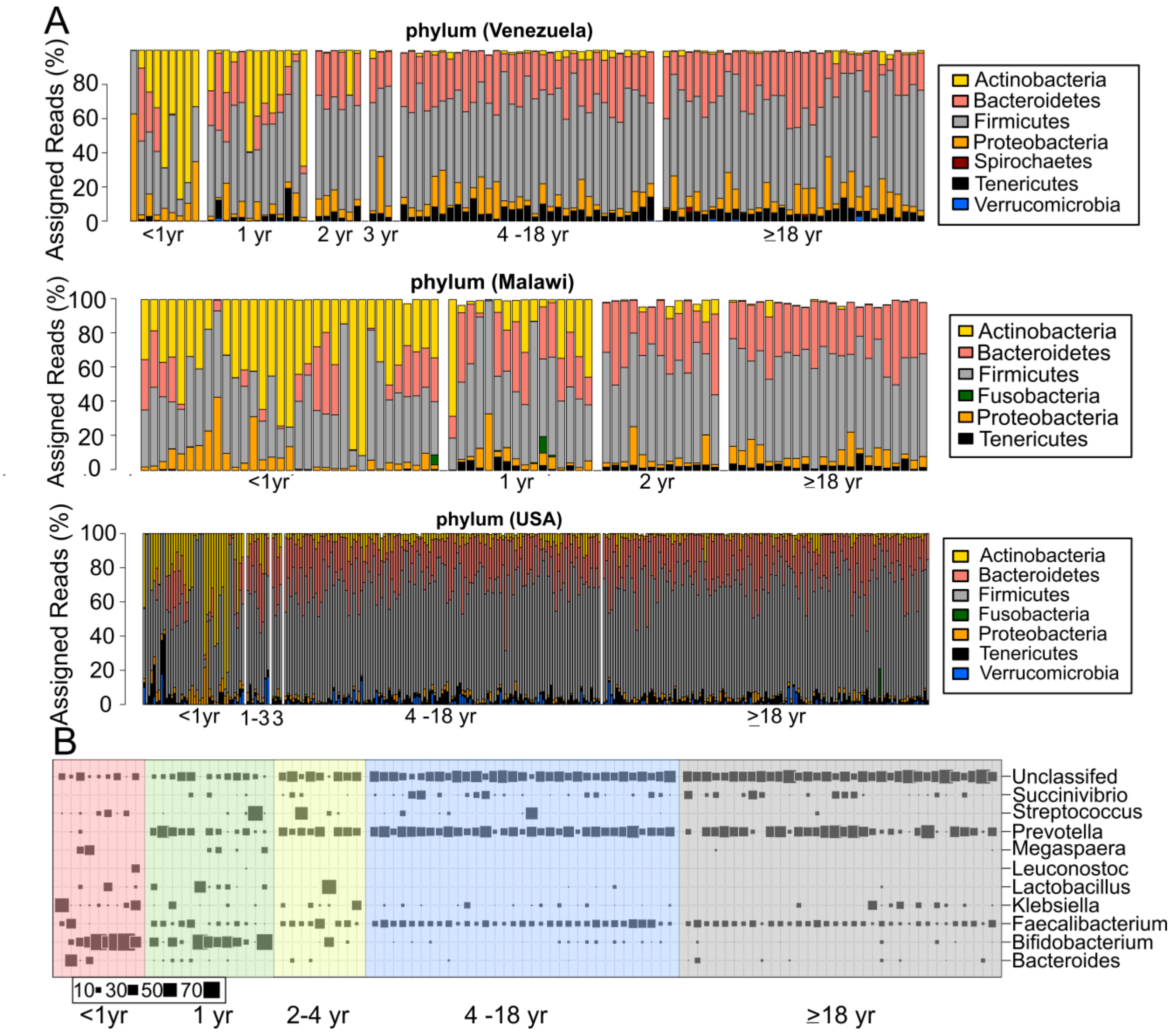

Figure S3: Visualization of the intestinal microbiota as (A) bar chart and (B) bubble plot. (A) Each bar represents the composition of the intestinal microbiota of one individual on rank phylum with a minimal relative abundance of 5% for the subjects from Venezuela, Malawi and USA. Subjects were grouped by age. (B) Columns represent individuals from Venezuela. Square sizes depict the relative abundance of each genus. Only the 10 most abundant genera are shown. Individuals were grouped by age. Age groups were manually colored by editing the exported svg file in Inkscape (https://inkscape.org/).

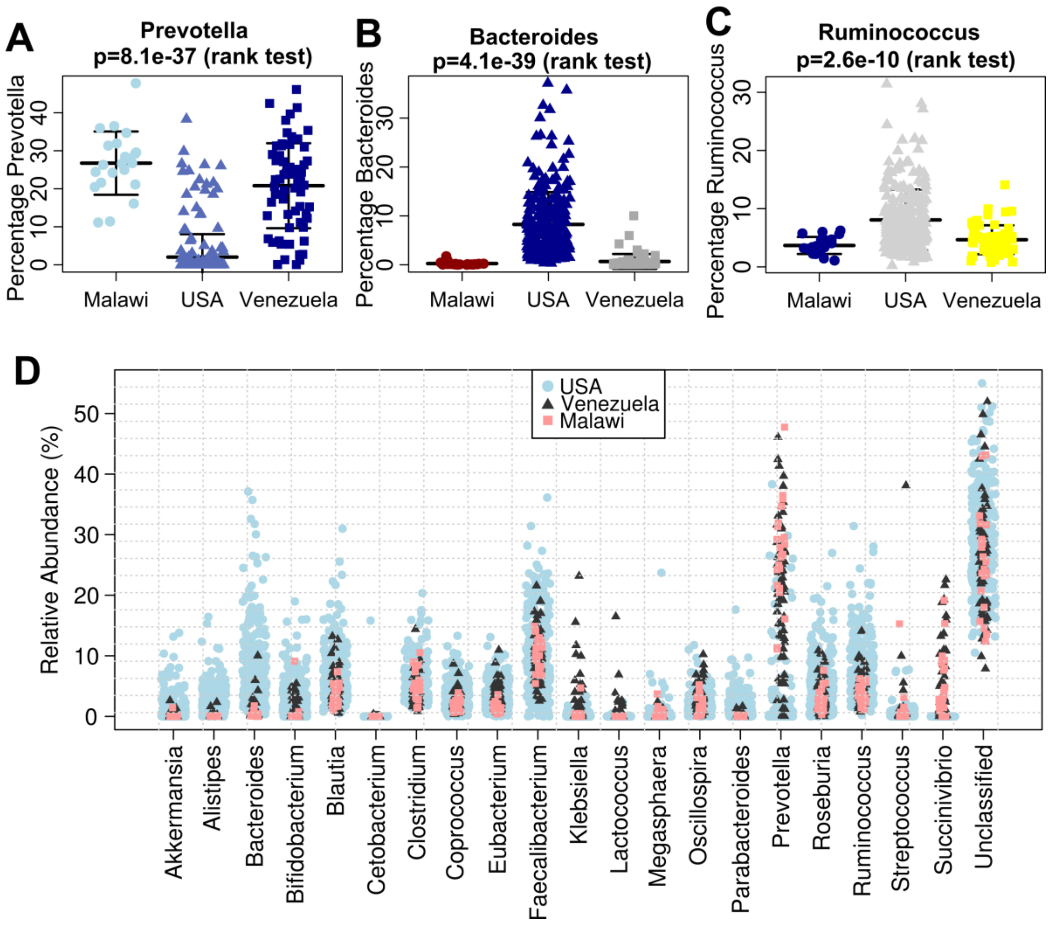

Figure S4: Geography associated differences in the intestinal microbiota using stripcharts, cluster and network analysis. (A) *Prevotella* showed a significantly different relative abundance in Malawi, USA and Venezuela ($p<10^{-36}$, Kruskal-Wallis test). (B). *Bacteroides* were significantly differently abundant between Malawi, USA and Venezuela ($p<10^{-38}$, Kruskal-Wallis test). (C) Prevalence of *Ruminococcus* was significantly different between Malawi, USA and Venezuela ($p<10^{-09}$, Kruskal-Wallis test). (D) Strip chart representing the relative abundance of the 20 most abundant bacterial taxa in non-infants (>3yr).

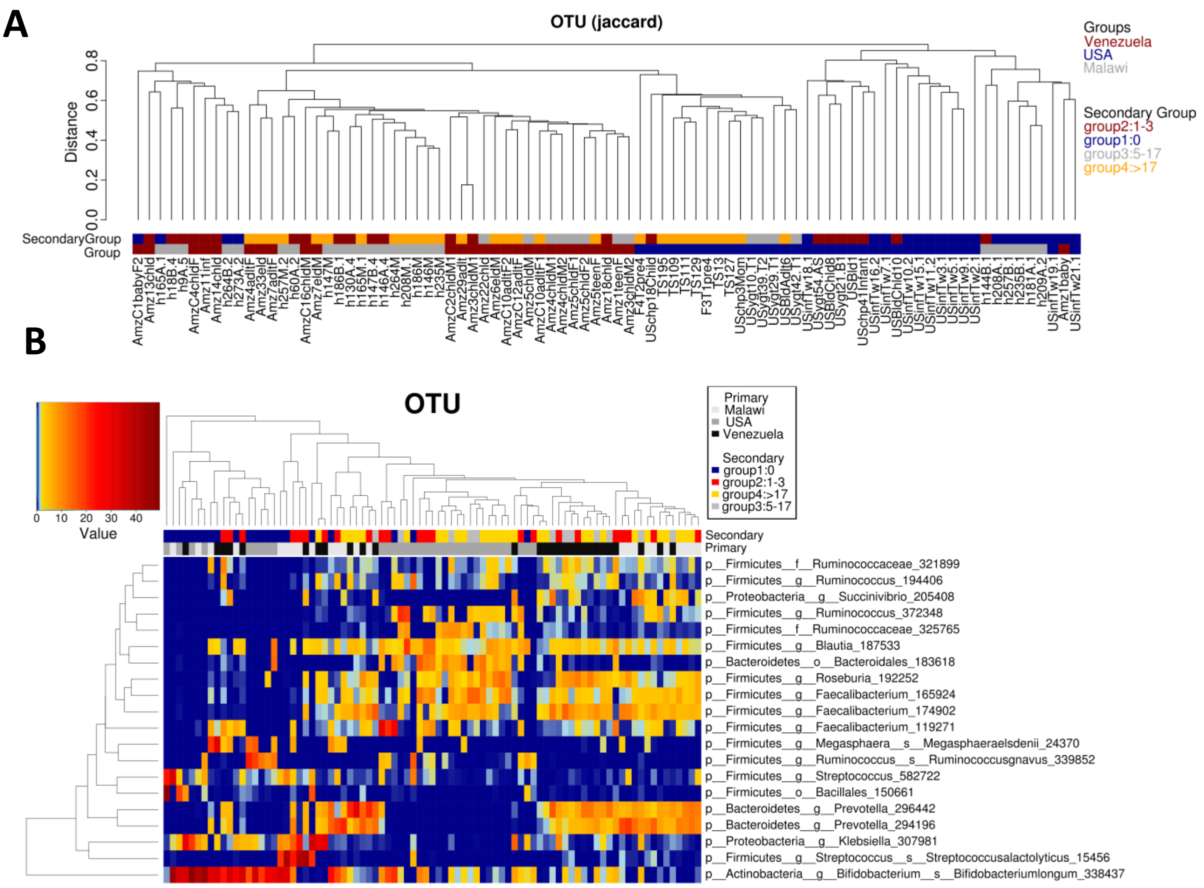

Figure S5: Cluster visualized as dendrogram or heatmap: (A) The dendrogram based on OTU level shows the relationship between the samples. Meta-information is displayed below the dendrogram. Samples from Venezuela and Malawi tend to form a cluster. (B) Heatmap of the taxonomic composition on OTU level. The columns represent the samples and rows the top 20 OTUs. The heatmap ranges from blue to red, where red indicates a high abundance and blue a low abundance of the OTUs in the sample. The samples are color coded by primary and secondary group as shown in the bars below the dendrogram. The samples cluster by age and by location.

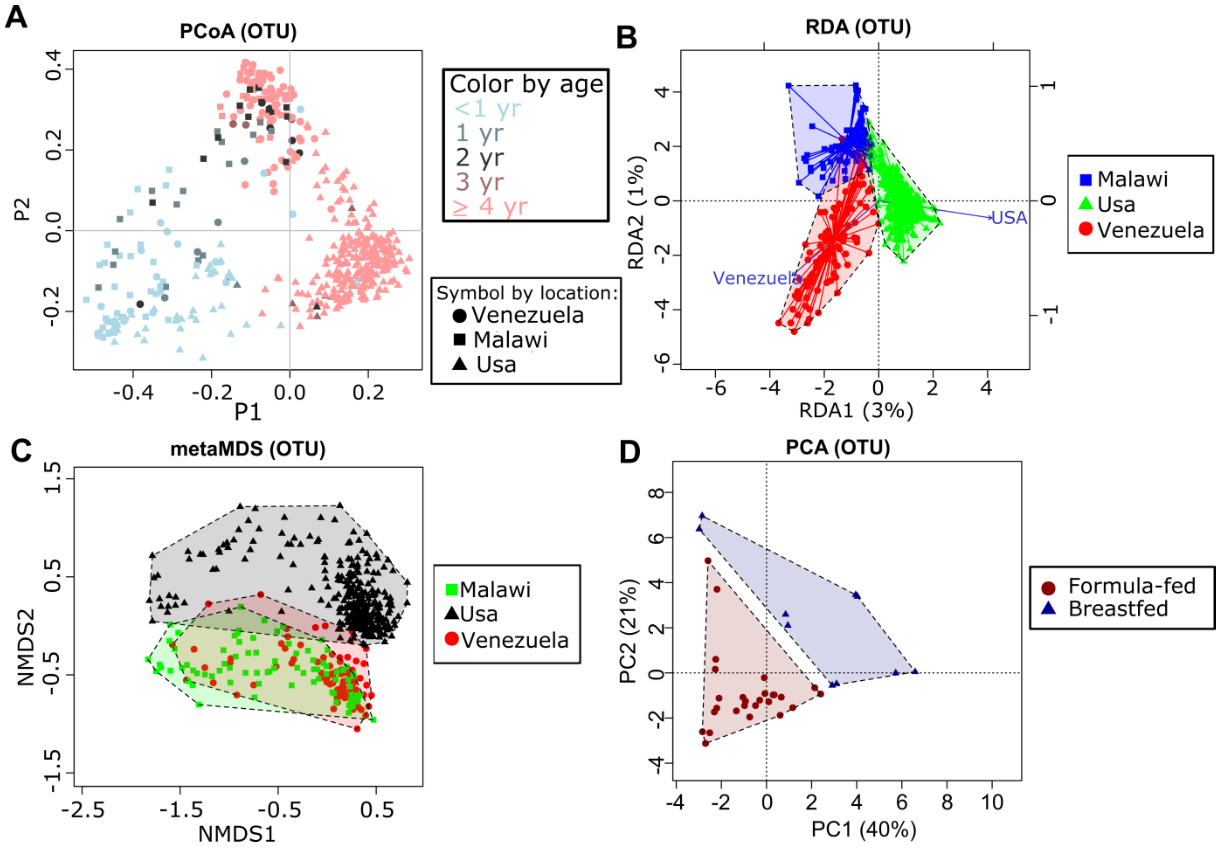

Figure S6: Multivariate analysis of the intestinal microbiota in respect to age and location. (A) PCoA of OTUs profiles compared by Jaccard distance. Colors indicate age ranges, symbols geographic location. Samples cluster by age and geography. (B) Nonmetric multidimensional scaling (NMDS) of OTUs profiles. Clusters of Venezuela and Malawi samples overlap, while USA forms a separate cluster. (C) Redundancy analysis (RDA), a supervised multivariate method for studying microbiome-environment associations. Both age and geographic location were significantly associated with fecal microbiota composition. RDA was run on genus profiles. (D) Principal component analysis (PCA) of OTU profiles of USA infants. Intestinal microbiota of breastfed and formula-fed infants formed distinct clusters.

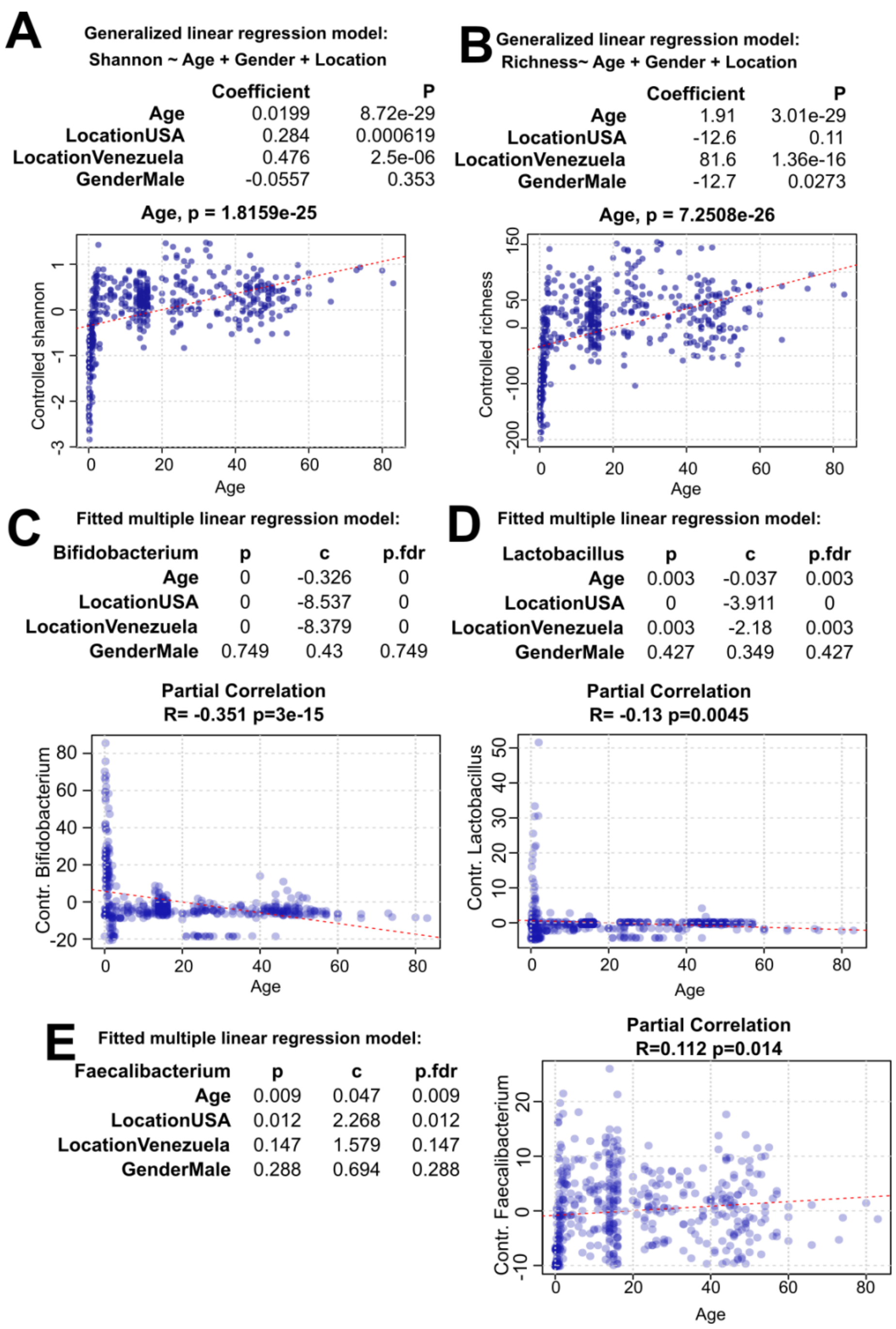

Figure S7: Multivariable regression identified complex associations between age, geography, and gender and community composition. (A) Linear multivariable regression including community diversity (Shannon index) as dependent variable and location, age and gender as explanatory variables. Community diversity was strongly associated with age ($p<10^{-28}$) and location ($p<10^{-5}$) but not gender ($p=0.35$). The scatterplot visualizes the positive correlation between age and microbial diversity adjusted for location and gender (partial correlation). (B) Richness was associated with age ($p<10^{-28}$), location and gender ($p=0.027$). The scatterplot visualizes the positive correlation between age and microbial richness adjusted for location and gender (partial correlation). (C) Association between age and relative abundance of

Bifidobacterium. The regression model included relative abundance of *Bifidobacterium* as dependent variable and age, location and gender as explanatory variables. The regression analysis identified a strong negative association between relative abundance of *Bifidobacterium* and age (FDR< 10^{-45}). The scatterplot depicts the negative association between *Bifidobacterium* and age adjusted for location and gender (partial correlation). (D) Association between age and relative abundance of *Lactobacillus* (FDR=0.003). The regression model included relative abundance of *Lactobacillus* as dependent variable and age, location and gender as explanatory variables. The scatterplot depicts the negative association between *Lactobacillus* and age adjusted for location and gender (partial correlation). (E) Association between age and relative abundance of *Faecalibacterium* (FDR=009). The regression model included relative abundance of *Faecalibacterium* as dependent variable and age, location and gender as explanatory variables. The scatterplot depicts the positive association between *Faecalibacterium* and age adjusted for location and gender (partial correlation).

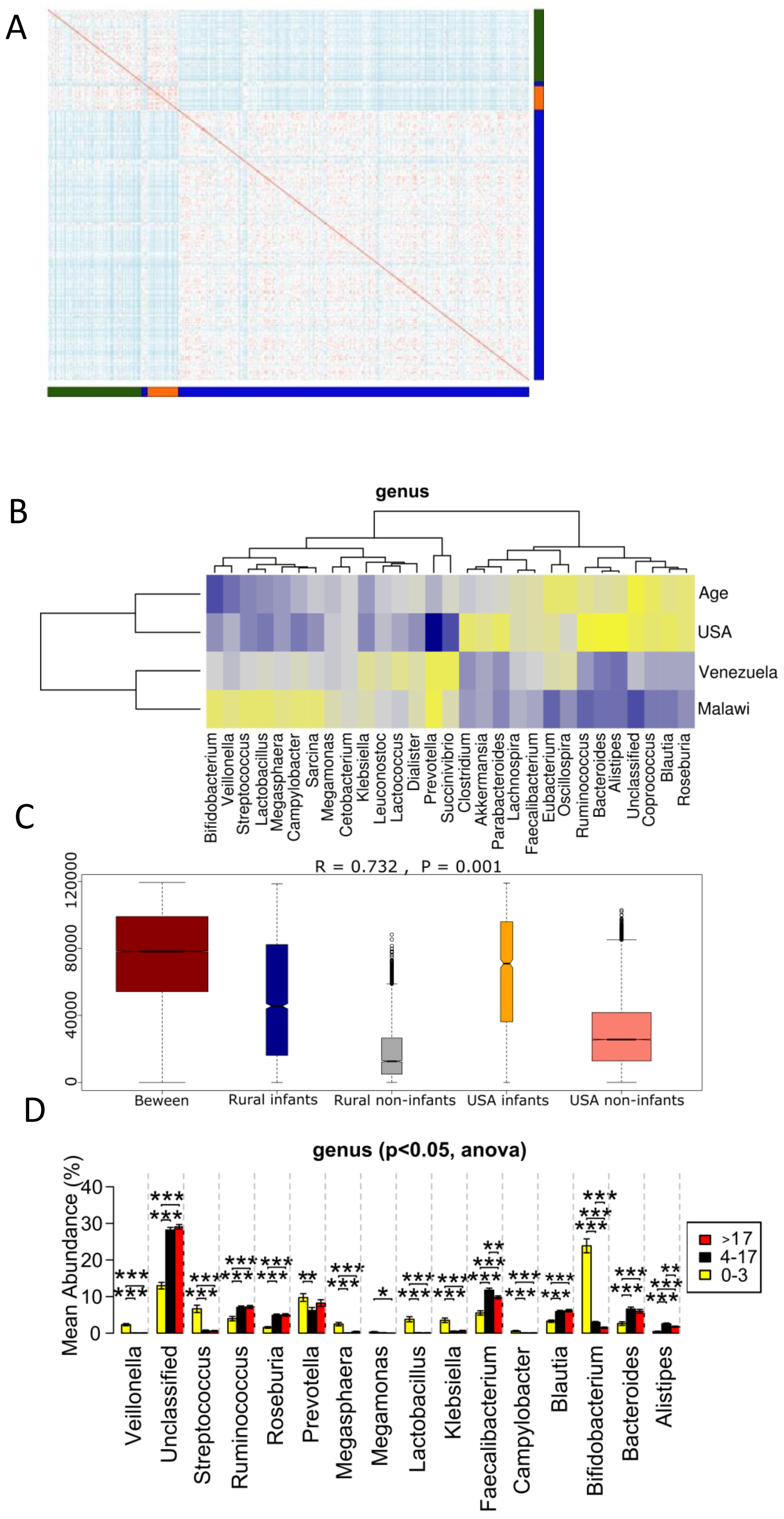

Figure 8: (A) Network analysis: Similarity of non-infant (>3 years) intestinal microbiota. Samples are color-coded by location (green: Venezuela; orange: Malawi; blue: USA). Pair-wise Jaccard distance of community composition profiles is represented in color code ranging

from red (highly similar) to blue (low similarity). Intestinal microbiota of Venezuela and Malawi populations shows a similar community structure, which is different from the intestinal microbiota of the USA population. (B) Heatmap visualizing Pearson’s correlation between relative abundance of bacterial genera with a minimal abundance of at least 2% and location and age. Positive correlations are shown in yellow, negative correlations in blue. (C) ANOSIM comparing intra-group and between-group distances of microbial community profiles at OTU level. Intra group distances were significantly smaller than between group distances ($p=0.001$), indicating that geographic location is associated with gut microbiota composition. The infant microbiota shows a higher variability (intra-group distance) when compared to adults. (D) Significantly differentially abundant microbial genera between age ranges 0-3, 4-17 and >17 years ($p<0.05$, Anova). p-values: *, <0.05; **, <0.01, ***, <0.001

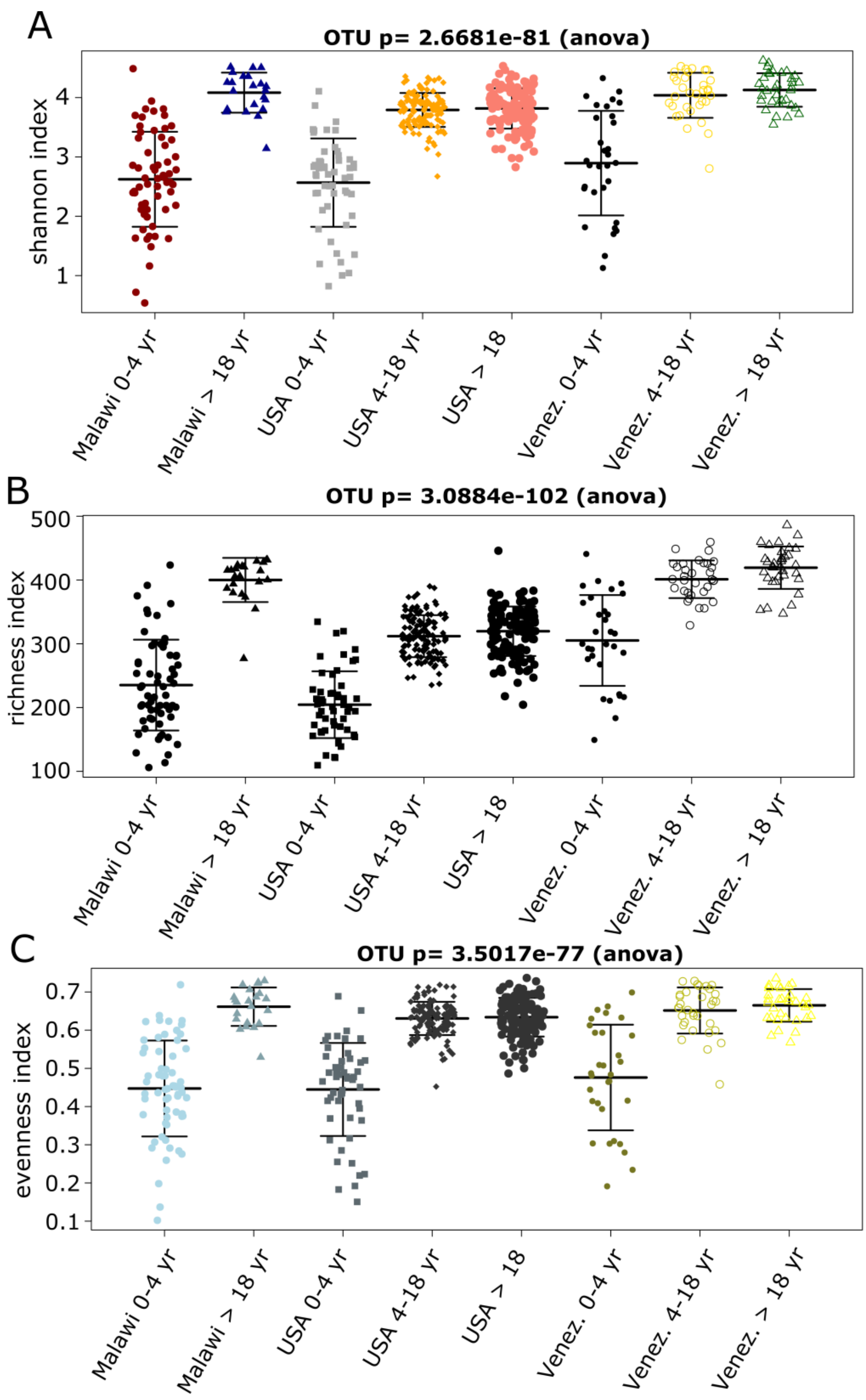

Figure S9: Microbial diversity, richness and evenness at 3 geographic locations and 3 age groups. Samples were grouped into age groups infants (≤3 years), children (3-17 years) and adults (>17 years). Diversity (Shannon index) (A), richness (B) and evenness (C) increase with age in all three populations. The microbial richness of the US population was lower than in Venezuelans and Malawians individuals.

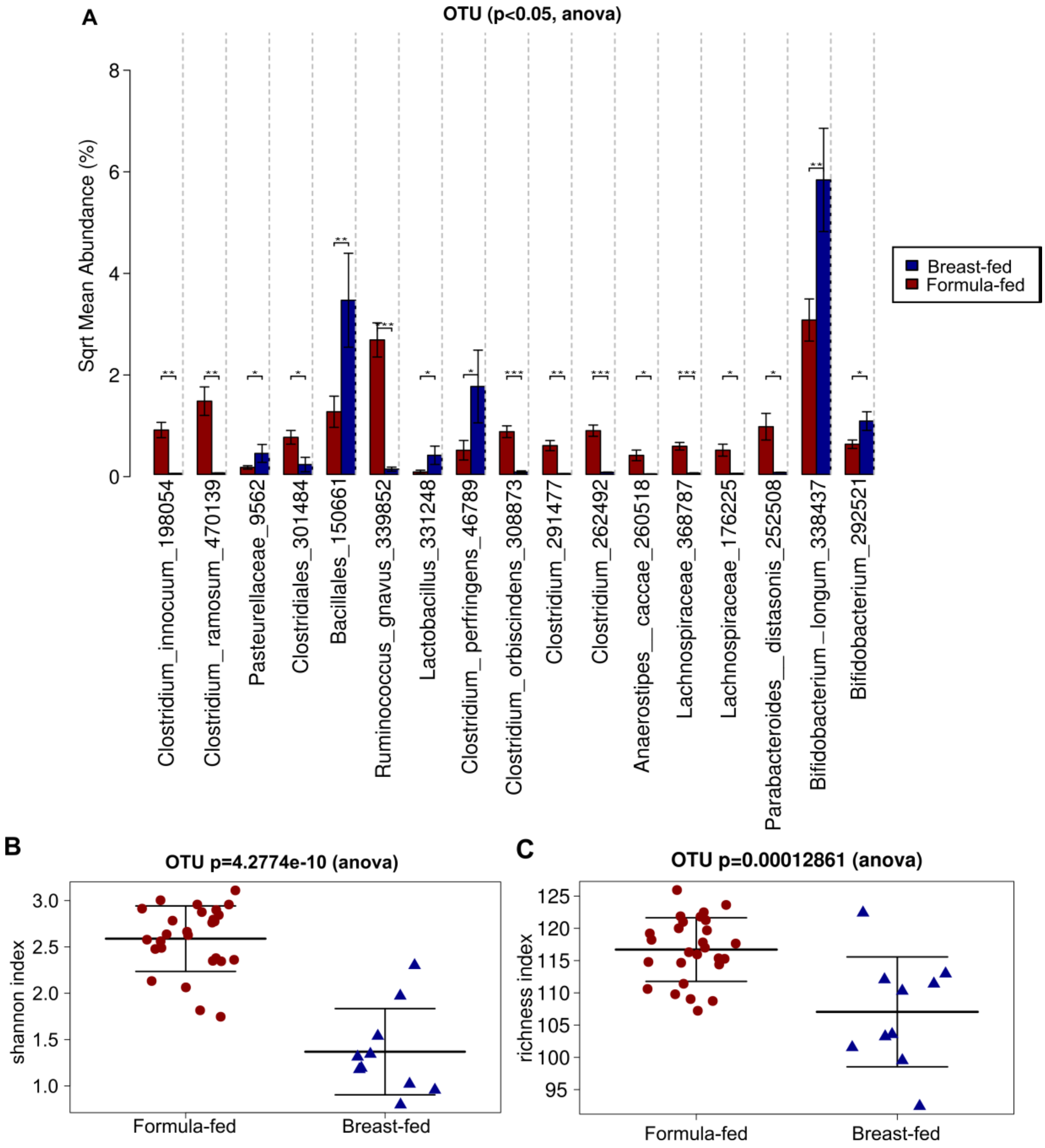

Figure S10: Changes associated with breastfeeding. (A) Significantly different OTUs between breast-fed and formula-fed infants in the USA cohort ($p<0.05$, ANOVA) p-values: *, <0.05; **, <0.01, ***, <0.001. (B) Microbial diversity measured by Shannon index of the fecal microbiota of breast-fed and formula-fed USA infants ($p<10^{-9}$, Anova). (C) Fecal microbial communities of breast-fed and formula-fed USA infants showed a significant different microbial richness ($p<10^{-3}$, Anova).

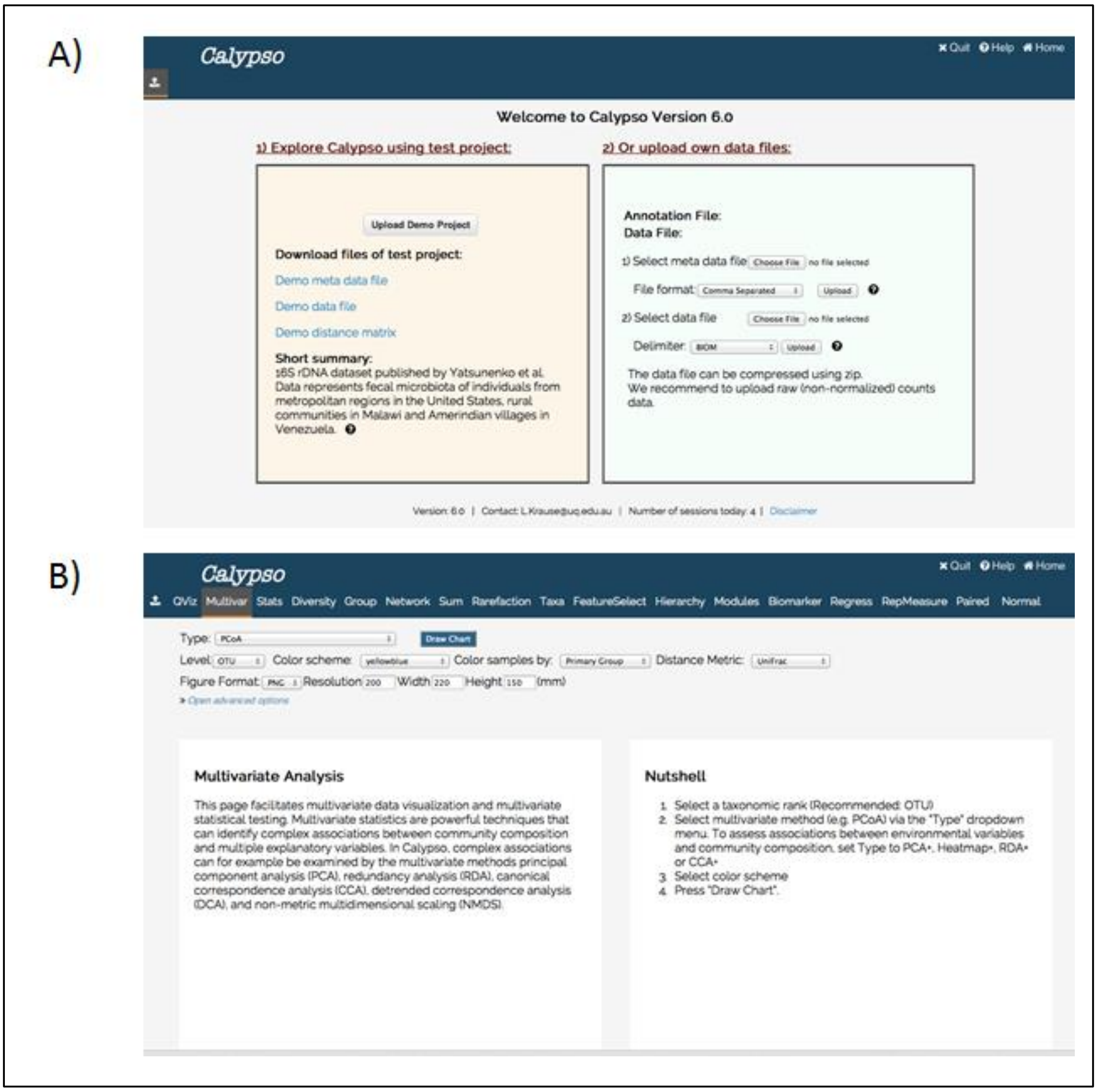

Figure S11: Calypso user interface. A) Data upload page. B) Multivariate tab to exemplify the design of the web-interface. A short description is provided on the bottom left of each page and a "Nutshell" section is presented on the bottom right. Advanced options are shown in "Advanced" menus. Tooltips provide short descriptions for most options.
